# Integrated network pharmacology and *in vivo* experiments to reveal the anti-inflammatory mechanism of Qinghuo Rougan Formula in uveitis

**DOI:** 10.3389/fmolb.2025.1632027

**Published:** 2025-07-11

**Authors:** Changying Jing, Yaqi Sun, Hongsheng Bi, Junguo Guo, Cong Ren, Jike Song, Beibei Wang, Qingmei Tian, Dadong Guo, Pengjuan He, Lijie Li, Xiaofeng Xie

**Affiliations:** ^1^ Shandong Provincial Key Laboratory of Integrated Traditional Chinese and Western Medicine for Prevention and Therapy of Ocular Diseases, Key Laboratory of Integrated Traditional Chinese and Western Medicine for Prevention and Therapy of Ocular Diseases in Universities of Shandong, Shandong Academy of Eye Disease Prevention and Therapy, Jinan, China; ^2^ Munich Medical Research School (MMRS), Klinikum der Ludwig-Maximilians-Universität München, Munich, Germany; ^3^ Medizinische Klinik und Poliklinik IV, Klinikum der Ludwig-Maximilians-Universität München, Munich, Germany; ^4^ Shandong Provincial Key Laboratory of Metabolic Diseases, Shandong Provincial Clinical Research Center for Immune Diseases and Gout, The Affiliated Hospital of Qingdao University, Qingdao, Shandong, China; ^5^ Department of Obstetrics and Gynecology, Maternal and Child Health hospotal of Shandong Province, Jinan, Shandong, China; ^6^ Endocrine and Metabolic Diseases Hospital of Shandong First Medical University, Shandong Institute of Endocrine and Metabolic Diseases, Jinan, Shandong, China

**Keywords:** uveitis, network pharmacology, WGCNA, Qinghuo Rougan Formula, systems biology

## Abstract

**Background:**

Uveitis is a complex intraocular inflammatory disease and pathology results from the continuous production of proinflammatory cytokines in the optical axis. Qinghuo Rougan Formula (QHRGF), a traditional Chinese medicine (TCM) is now used to treat uveitis with desirable effect. However, the mechanism of action is still unclear. This study aimed to explore the potential diagnostic and therapeutic biomarkers for uveitis using systems biology methods, including network pharmacology and weighted gene co-expression network analysis (WGCNA).

**Methods:**

A molecular drug-compound-target-uveitis interaction network was established using network pharmacology. Functional enrichment analyses were performed to screen potential signaling pathways. The uveitis gene expression dataset from the Gene Expression Omnibus database was subjected to WGCNA to identify gene co-expression modules related to uveitis and explore the potential hub genes. The least absolute shrinkage and selection operator (LASSO) model was used to identify the hub genes. Additionally, molecular docking was performed to verify the accuracy and stability of the model. Finally, the suppressive effects of QHRGF on uveitis were experimentally verified *in vivo*.

**Results:**

Network pharmacology and functional enrichment analysis showed that 18 targets and immune/inflammation-related pathways were associated with the QHRGF-targeted pathway network. The yellow module contained 120 genes had a strong correlation with uveitis using WGCNA. In total, 12 putative targets of QHRGF, differentially expressed genes, and yellow module genes were determined. Six hub genes were identified using LASSO model and the receiving operating characteristic curve analysis demonstrated the model can serve as biomarkers for uveitis. The advantages of these genes were approved using molecular docking. Finally, *in vivo* experiments provided evidence confirming that QHRGF was identified as the key target of the anti-inflammatory effect of uveitis.

**Conclusion:**

In conclusion, this research revealed that QHRGF can be used to treat uveitis through multiple components and targets. Meanwhile, the potential anti-inflammatory action of QHRGF in the treatment of uveitis was verified by combining network pharmacology and *in vivo* experiments, suggesting its potential as a quite prospective agent for the therapy of uveitis.

## 1 Introduction

Uveitis, which is an intraocular inflammatory disorder in developed countries, can be classified according to the parts of the eye involved (anterior, intermediate, posterior, or panuveitis) or etiology (infectious or non-infectious) (Caspi, 2010; Santeford et al., 2016). Conventional treatment for non-infectious uveitis is non-specific and includes the frequent use of topical and/or systemic corticosteroids and other immunosuppressive agents or biologics, such as anti-tumor necrosis factor-α (TNF-α) antibodies. However, these therapeutic strategies do not effectively prevent uveitis relapse (Crabtree et al., 2019). Mounting studies have shown that uveitis is associated with bacterial, viral infections, genetic, and autoimmune factors (Chen et al., 2005; Klaska et al., 2017). Targeted immunotherapy is an effective treatment strategy for autoimmune disease (Daveson et al., 2017) and the therapeutic potential of targeted immunotherapy for uveitis has piqued the interest of the scientific community. Thus, there is an urgent need to identify novel promising therapeutic avenues, the underlying mechanism of pathogenesis, or novel immune-related biomarkers with increased specificity to facilitate early diagnosis and establish a comprehensive therapeutic schedule.

The main aim of systems biology, a biological science field, is to predict the system-level biological networks and molecular interactions (Danchin, 2009). The elucidation of molecular interactions, such as protein-protein interactions (PPIs) and protein-small molecule interactions are critical to explore the mechanism of biological processes and identify treatments for diseases (Balaji et al., 2012). Network pharmacology is a commonly applied strategy that enables to comprehensively understand the complex relationship between drugs and diseases based on the interaction among drugs, ingredients, targets, and diseases (Hopkins, 2008). Recently, traditional Chinese medicine (TCM)-based therapeutics or natural medicines have become increasingly popular owing to their advantages of multi-ingredient, multi-link, and multi-target principles (Wang et al., 2008). Therefore, network pharmacology or system biology can provide a novel strategy to elucidate the molecular interactions between bioactive components and the underlying mechanisms of TCM from a systemic and holistic perspective.

Qinghuo Rougan Formula (QHRGF), a therapeutic used in TCM, has been widely used to treat uveitis for several decades. The composition of QHRGF is as follows form. Previously, we examined the 10 major herbal components of QHRGF and demonstrated that QHRGF exerts potent immunomodulatory effects and decreases the occurrence of uveitis (Jing et al., 2019). In this study, a molecular interaction network was established for the active small molecule compounds of QHRGF and their protein targets using network pharmacology. The therapeutic targets of QHRGF for uveitis were predicted. The uveitis gene expression data were retrieved from the Gene Expression Omnibus (GEO) database to identify co-expression modules related to the disease status using weighted gene co-expression network analysis (WGCNA). Furthermore, the potential hub genes were identified, and a prognostic model was constructed to distinguish uveitis from health. Molecular docking was then performed to verify the accuracy and stability of the model. Finally, we carried out biological experiments to validate the mechanism by which QHRGF mediates its therapeutic effect on uveitis. This research shifted the focus from simple network pharmacological analysis to the mathematical modeling of the systems biology approach, which improved our understanding and enabled the prediction of the molecular mechanisms underlying uveitis.

## 2 Methods

### 2.1 Preparation of QHRGF decoction

The composition of QHRGF was listed in Supplementary Table S1. There were 13 medicinal ingredients including Gentian and others, all of which were purchased from Shandong Baiwei Tang Traditional Chinese Medicine Decoction Pieces Co., Ltd. Each bag of this product was measured in terms of gentiopicroside (C_16_H_20_O_9_), which should not be less than 4.2 mg, and the content of gardenia with geniposide (C_17_H_24_O_10_) and Scutellaria baicalensis with baicalin (C_21_H_18_O_11_) should not be less than 4.2 mg and 13.8 mg, respectively. The ingredients were decocted twice, the first time with 10 times quantity of water for 2 h, and the second time with 8 times quantity of water for 1 h. The combined filtrates were concentrated under reduced pressure at 60°C to a relative density of 1.5 g/mL. Then, the cornstarch was incorporated into the slurry and mixed well, granulated, dried, shaped, and packed.

### 2.2 High-performance liquid chromatography (HPLC)

For quantitative analysis, a mixed reference solution of the standards (30 μg gentiopicroside, 30 μg gardenoside, and 60 μg baicalin) were prepared by precisely weighting and dissolving them in 1 mL methanol. The QHRGF sample (2.0 g) was weighed accurately, grinded, and extracted by ultrasonication with 50 mL of 50% methanol for 30 min, then the solution was stirred for homogeneity and filtered. The stationary phase was octadecylsilane-bonded silica gel, and the mobile phase A was acetonitrile, and the mobile phase B was 0.1% formic acid solution. The detection wavelength was 254 nm. The number of theoretical plates of the baicalin peak should be no less than 2,000. Precisely extract 5 μL of the reference solution and the sample solution respectively, inject them into the liquid chromatograph for measurement.

### 2.3 Selection of target compounds of QHRGF and prediction of targets

The compounds of the 13 herbs of QHRGF were downloaded from the Traditional Chinese Medicine Systems Pharmacology (TCMSP, Version 2.3, http://lsp.nwu.edu.cn/) database (Ru et al., 2014). The candidate active ingredients were screened based on the following criteria using the *in silico* absorption, distribution, metabolism, and excretion integrative model: oral bioavailability (OB) ≥ 30%; drug likeness (DL) ≥ 0.18. OB is a pharmacokinetic parameter that estimates the percent of an orally administered drug reaching systemic circulations. Meanwhile, DL is a qualitative concept used in drug design to estimate compounds with “drug-like” properties (Pang et al., 2018). Additionally, the predicted target genes of TCM ingredients were obtained from the Encyclopedia of Traditional Chinese Medicine (ETCM, http://www.tcmip.cn/ETCM/) database (accessed on March, 2024), which is a comprehensive data resource that aids in the mechanistic investigation, new drug discovery, and clinical application of TCM (Xu et al., 2019). The two databases can compensate each other for the lack of some data on compounds.

### 2.4 Identification of uveitis-related and immune-related genes

The uveitis-related target genes were obtained from the GeneCards database (https://www.genecards.org/, accessed on March, 2024), which is a searchable, integrative database that furnishes information of all annotated and predicted human genes (Qian et al., 2020). The keywords “uveitis” and “immune” were used to search for uveitis-related and immune-related targets, respectively.

### 2.5 Microarray data collection and procession

In this study, the GSE7850 dataset (https://www.ncbi.nlm.nih.gov/geo/query/acc.cgi?acc=GSE7850), comprising data of uveitis and healthy (control) samples, was selected by narrowing the study type and organism to “expression profiling by array” and “*Homo sapiens*,” respectively, as well as using the inclusion criteria. The gene expression profile of GSE7850, containing data on 24 uveitis and 20 healthy samples, submitted by Justine Smith et al. was analyzed using the GPL201 platform (Affymetrix Human HG-Focus Target Array). The probe name was converted to gene symbols using the platform annotation information and probes with missing expression values were removed. The average values of genes with multiple corresponding probes were used as the expression values.

### 2.6 Identification of differentially expressed genes (DEGs)

The Bayesian method of the Linear Models for Microarray (Limma) package in R software was applied for identifying DEGs between uveitis and healthy tissues (Diboun et al., 2006). The gene expression data from the GSE7850 dataset were log2-transformed and quantile normalized prior to differential expression analysis using the limma package. The *P*-values were adjusted for multiple testing correction with the Benjamini–Hochberg method to control the false discovery rate (FDR). Significant DEGs were identified based on the following criteria: |log_2_ fold change (FC)| > 1 and adjusted P < 0.05. The DEGs of the GSE7850 dataset were visualized using the volcano plot and heatmap, which were constructed using the R package ‘ggplot2.’

### 2.7 Network construction and central network topological analysis

To comprehensively understand the molecular mechanisms of uveitis, the drug-compound-target-disease interaction network was constructed for targets of QHRGF and DEGs based on the interaction data. The network was visualized using the Cytoscape software (Version 3.9.2, http://www.cytoscape.org/). In this network, nodes represented the TCM-based therapeutics, compounds, or targets, while the edges represented the compound-target interactions.

The central network analysis was performed using the topological method with the Cytoscape software plugins CytoNCA and BisoGenet (Tang et al., 2015). BisoGenet provides an easy-to-use interface that allows users to customize searches by specifying a target set of genes to retrieve their molecular interactions from an in-house database of Cytoscape (Martin et al., 2010). Two topological properties (degree centrality, DC and betweenness centrality, BC) were calculated to analyze the central topological attributes of the nodes in the network. The levels of these two parameters represent the topological importance of the nodes in the network. In addition to analyzing the interactions among the target genes, other genes associated with the target genes were examined to accurately identify the hub targets.

### 2.8 Functional enrichment analysis

Gene Ontology (GO) and Kyoto Encyclopedia of Genes and Genomes (KEGG) pathway enrichment analyses were used to explore the underlying biological progresses and functional pathways of the targets with the clusterProfiler v3.6.0 (Yu et al., 2012). Enrichments were considered significant at adjusted *P* < 0.05.

### 2.9 Weighted gene co-expression network analysis (WGCNA)

Weighted gene co-expression network analysis (WGCNA) was performed to identify gene modules associated with uveitis using the WGCNA package in R (Langfelder and Horvath, 2008). Expression data from the GSE7850 dataset were used as input. Outlier samples were removed based on hierarchical clustering. A soft-thresholding power of β = 5 was selected to achieve a scale-free topology (*R* ^2^ > 0.9). An adjacency matrix was constructed and transformed into a topological overlap matrix (TOM), followed by module detection using dynamic tree cutting with a minimum module size of 50 genes. Modules with similar expression profiles were merged using a threshold of 0.25. Module eigengenes (MEs) were calculated as the first principal component of each module. The correlation between MEs and uveitis status was assessed using Pearson correlation analysis to identify disease-relevant modules. The module most strongly associated with uveitis was selected for downstream analysis.

### 2.10 Identification of overlapping genes

Venn diagram, which was drawn using the R package ‘Venn Diagram,’ was constructed to obtain the overlapping genes among the target genes of active ingredients, DEGs, and co-expression genes. Analyzing the functional interactions between proteins can provide novel insights into the function of proteins and improve our understanding of the general organizing principles of functional cell systems. To mine the data of the direct or indirect regulatory relationship, a PPI network was generated from Search Tool for the Retrieval of Interacting Genes (STRING) database (version 11.5; https://string-db.org/). Cytohubba, which was used to analyze the genes, is a Cytoscape plug-in that can be used in 11 calculation methods to analyze and discover key targets and subnetworks of a complex network (Chin et al., 2014). Additionally, principal component analysis (PCA) was employed to investigate the difference between uveitis and healthy samples using the overlapped gene expression profiles.

### 2.11 Identification of hub genes using least absolute shrinkage and selection operator (LASSO)

LASSO was used to further narrow down the range of genes and obtain an optimal model with the lowest expected prediction error that can accurately predict observations in future sample analysis (Sohn et al., 2009). In this study, the expression profile of the overlapped genes was used to construct the LASSO model to distinguish uveitis samples from control samples. To select the optimal regularization parameter (λ), we performed five-fold cross-validation using the glmnet package in R (Friedman et al., 2010). The value of λ that minimized the cross-validated mean squared error was chosen to build the final model. The lambda selection curve is provided in Figure 5A. Given the limited sample size (n = 44), we did not split the data into training and test sets. Instead, cross-validation was used to avoid overfitting and ensure model robustness. Finally, several hub genes were retained to construct the prognostic model. A model index of individual sample was calculated as follows:

$$\text{Risk index}=\sum Coe\left(i\right)\times x\left(i\right)$$

where, *Coef*(*i*) and *x*(*i*) indicate the estimated regression coefficient from LASSO analysis and the expression value of each hub gene, respectively. The receiver operating characteristic (ROC) curve and the area under the ROC curve (AUC) were calculated to examine the accuracy of the constructed signature predictions.

### 2.12 Gene set enrichment analysis (GSEA)

GSEA was used to screen potential KEGG pathways based on the overlapped gene expression profiles (Subramanian et al., 2005). The c5.all.v7.1.symbols.gmt and c2.cp.kegg.v7.1.symbols.gmt datasets in the MsigDB V7.1 database were used as reference gene sets (Liberzon et al., 2015). These pre-ranked genes were analyzed using GSEA with GSEA software (http://www.broadinstitute.org/gsea) using default parameters. Enrichments were considered significant at *P* < 0.05 and FDR <0.25.

### 2.13 Molecular docking

The 2D structure of the active ingredients corresponding to the hub genes was obtained from the PubChem database (https://pubchem.ncbi.nlm.nih.gov/). Next, the crystal structure file of the protein was downloaded from RCSB Protein Data Bank (RCSB PDB) database (http://www.rcsb.org/pdb/home/home.do). Molecular docking was carried out using AutoDock Vina (Version 1.2.0) (Trott and Olson, 2010). The 2D structures of active compounds were retrieved from PubChem, and protein crystal structures were downloaded from the RCSB Protein Data Bank. Docking poses were ranked by binding energy (kcal/mol). For each compound–target pair, we selected the pose with the lowest docking score. These top-ranked conformations were visualized using PyMol (Version 3.03) (Lilkova et al., 2015).

### 2.14 Animals and induction of experimental autoimmune uveitis (EAU)

All animal experiments were carried out in accordance with the Committee guidelines of the Eye Institute of Shandong University of Traditional Chinese Medicine (2015-XK-013) and the Association for Research in Vision and Ophthalmology (ARVO) Statement for the Use of Animals in Ophthalmic and Vision Research. All surgeries were performed under anesthesia, and all efforts were made to minimize animal discomfort and stress. Female Lewis rats aged six to 8 weeks and weighing 160–180 g were purchased from Beijing Vital River Laboratory Animal Ltd. (Beijing, China). Prior to the study, all the rats were adapted to the housing conditions for 7 days. Meanwhile, a routine examination was performed on all subjects to rule out pre-existing eye diseases. The experimental conditions for the animals were as follows: controlled room temperature of 25°C ± 1.731°C, a relative humidity of 50% ± 10%, and a 12-h light/dark cycle.

Healthy Lewis rats aged 6–8 weeks were randomly divided into the following groups: healthy control (NC) (n = 30), EAU (n = 30), and QHRGF groups (n = 30). An emulsion of interphotoreceptor retinoid-binding protein (IRBP) was prepared by dissolving 100 µg of IRBP peptides, 100 µg of *Mycobacterium tuberculosis* H37Ra (strain H37 Ra), and 150 µL of complete Freund’s adjuvant (CFA) in sterilized phosphate-buffered saline (PBS, pH = 7.2) and making up the volume to 300 µL. On day 0, a total of 300 µL of IRBP emulsion was subcutaneously injected in three sites: base of tail and both thighs (EAU and QHRGF groups). The control rats were immunized only with CFA and H37RA.

### 2.15 Reagents and intervention with QHRGF

IRBP peptides (residues 1,177–1,191; sequence ADGSSWEGVGVVPDV) were synthesized by Shanghai Sangon Biological Engineering Technology & Services Co., Ltd. (Shanghai, China). H37Ra was purchased from Difco (Detroit, MI, USA), and CFA was purchased from Sigma-Aldrich (St. Louis, MO, USA). PBS, formaldehyde, paraffin, and hematoxylin and eosin (HE) stain were purchased from Sinopharm Chemical Reagent Co., Ltd. (Shanghai, China). Roswell Park Memorial Institute-1640 medium was purchased from Gibco; Thermo Fisher Scientific, Ltd. (Waltham, MA, USA). The rats in the QHRGF group were treated through oral gavage (1,000 mg/kg bodyweight/day) during the experimental period. Consecutive QHRGF was administered daily until the rats were sacrificed. The equal volume of sterilized PBS was used for NC and EAU groups.

### 2.16 Clinical evaluation and histopathological analysis

The rats were examined on days 0, 3, 5, 7, 9, 11, 13, 15, 17, 19, and 21 post-immunizations by Genesis-D camera (Kowa Company Ltd., Japan) for the evaluation of the clinical scores. The degree of inflammation was scored (Agarwal et al., 2012) on a scale of 0–4: Grade 0 indicates normal retinal architecture with no signs of inflammation. 0.5 reflects mild inflammatory cell infiltration affecting less than one-quarter of the section, with or without photoreceptor damage. Grade 1 to 4 represent increasing severity of inflammation and structural disruption, from photoreceptor outer segment damage to full-thickness retinal damage.

Rats in the NC, EAU, and QHRGF groups were randomly distributed on day 13 post-immunization, with 3 rats in each group. Rats were anesthetized and euthanized by intraperitoneal injection of 3% pentobarbital sodium (50 mg/kg).The eyeball was enucleated immediately and fixed in eyeball fixative solution (Servicebio, Wuhan, China) for 24 h at room temperature, The samples were then dehydrated, embedded, sectioned, and subjected to HE staining. The histopathological changes of the retina and ciliary body were observed under microscope (Ti; Nikon Corporation, Tokyo, Japan).

### 2.17 Quantitative-PCR (Q-PCR)

Total RNA was isolated from the eye tissues, spleen tissues and lymph nodes on day 13 post-immunization, and cDNA was synthesized from total RNA using the High-Capacity cDNA Reverse Transcription Kit (Thermo Fisher, USA). All qPCR reactions were performed on the LightCycler® 96 system (Roche Diagnostics) using the Fast SYBR Green Master Mix (Roche). Relative quantification was calculated by the 2^−ΔΔCT^ method and normalized by β-actin. The primer sequences for the target (IL-10, IL-4, IL-17, and IFN-γ) and reference genes primer are shown in Supplementary Table S2. Three biological replicates were used per group (n = 3).

### 2.18 Enzyme-linked immunosorbent assay (ELISA)

On day 13 post-immunization, extracted eye tissues, spleen tissues and lymph nodes from the three groups were ground with liquid nitrogen until reaching a uniformly fine powder, followed by the addition of TRI reagent. After sonication and incubation for 20 min on ice, extracts were centrifuged at 10,000 *g* at 4 °C for 20 min. The protein levels of IL-10, IL-4, IL-17, and IFN-γ on day 13 post-immunization were detected by ELISA using a multifunctional microplate reader. Three biological replicates were used per group (n = 3).

## 3 Results

### 3.1 High-performance liquid chromatography (HPLC)

**FIGURE 1:**
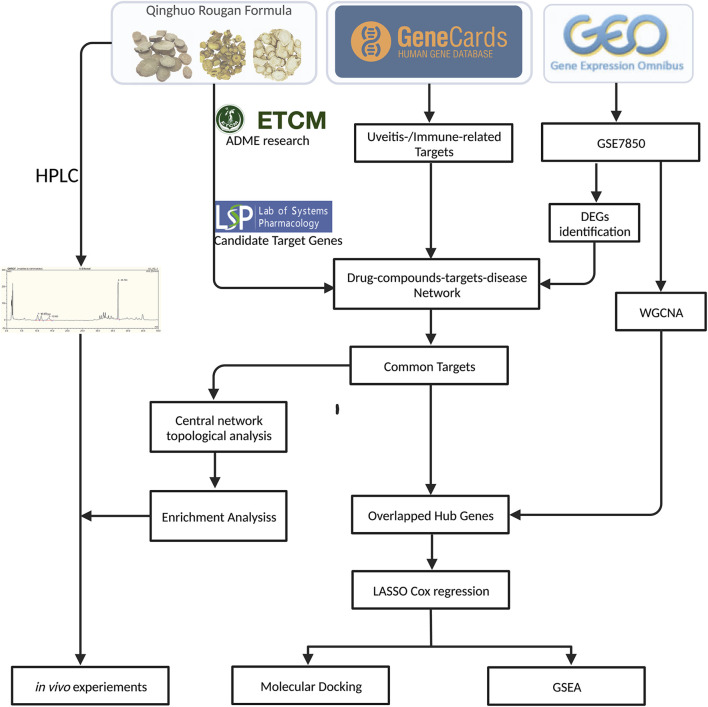
Overview of the study workflow. The study began with compound screening of Qinghuo Rougan Formula (QHRGF) using the TCMSP and, ETCM databases. Putative targets were integrated with uveitis-related genes from public databases. Differential expression analysis and weighted gene co-expression network analysis (WGCNA) were performed on the GSE7850 dataset to identify key disease-associated modules. Hub genes were selected using LASSO regression modeling. Molecular docking was used to assess compound-target binding. *In vivo* experiments in the EAU rat model were used to validate the anti-inflammatory effects of QHRGF.

Figure 1 shows the experimental workflow. The chemical composition of QHRGF was analyzed using high-performance liquid chromatography (HPLC). Many distinct peaks appeared in the chromatogram. Each peak represented a different compound (Supplementary Figures S1A–E). To identify the main active ingredients, we compared the peaks in the QHRGF extract with those of known reference standards. All samples were tested under the same chromatographic conditions. We confirmed three key compounds by matching their retention times. Baicalin showed a retention time of 7.84 min. Geniposide appeared at 9.52 min. Gentiopicroside appeared at 11.36 min. Their relative peak areas were 21.3%, 15.7%, and 12.9%. The peak for baicalin had more than 2000 theoretical plates. The resolution between peaks was greater than or equal to 1.5. These values showed that the compounds were well separated. We confirmed that the peaks matched the standard compounds. The reference standards were obtained from the National Institutes for Food and Drug Control (Beijing, China). The batch numbers are listed in Supplementary Table S1. These results showed that the QHRGF extract had stable and consistent chemical composition. This extract was used in both the computational and experimental parts of this study.

To validate the analytical method, we conducted a series of quality assessments. Specificity was confirmed by comparing QHRGF samples with negative controls lacking each individual component, which showed no interfering peaks at the respective retention times. The linearity was established over appropriate concentration ranges with excellent correlation coefficients: gentiopicroside (6.66–66.66 μg/mL, *R* ^2^ = 0.9989), geniposide (6.176–61.76 μg/mL, *R* ^2^ = 0.9948), and baicalin (13.334–133.34 μg/mL, *R* ^2^ = 0.9999) (Supplementary Figure S1F). Precision was supported by six repeated injections, with RSD values below 2%. Reproducibility was confirmed in six independently prepared sample batches, with RSDs ranging from 1.9% to 2.3%. Stability tests showed that all three compounds remained chemically stable at room temperature over a 24-h period, with peak area RSDs under 3.1%. These results confirm that the HPLC method used for QHRGF is specific, linear, precise, reproducible, and stable, and provides a reliable basis for qualitative and quantitative analysis.

### 3.2 Screening of active components of QHRGF and predicting their putative targets

In this study, a multi-dimensional analysis of the potential biomarkers of uveitis and targets of QHRGF in uveitis was performed using the integrated bioinformatics approach. To identify the active ingredients of QHRGF, the components of each herb in QHRGF were retrieved from the TCMSP database. Based on the screening criteria (OB ≥ 30% and DL ≥ 0.18), 117 active compounds were identified from 13 herbs of QHRGF (Supplementary Table S3). A part of the compound ‘MolName’ in the TCMSP database was missing, which was retrieved using the, ETCM database. In total, 3,520 potential targets of active components were identified and used for subsequent analysis (Supplementary Table S4).

### 3.3 Identification of immune-related genes and DEGs

The uveitis-related/immune-related targets were retrieved from the GeneCards database. In total, 1,033 uveitis-related were obtained. The GSE7850 dataset was downloaded from the GEO database. In total, 216 DEGs (170 upregulated genes and 46 downregulated genes) between uveitis and healthy tissues were identified. The DEGs were visualized using a heatmap (Supplementary Figure S2A) and a volcano plot (Supplementary Figure S2B).

### 3.4 Network construction and central network topological analysis

**FIGURE 2:**
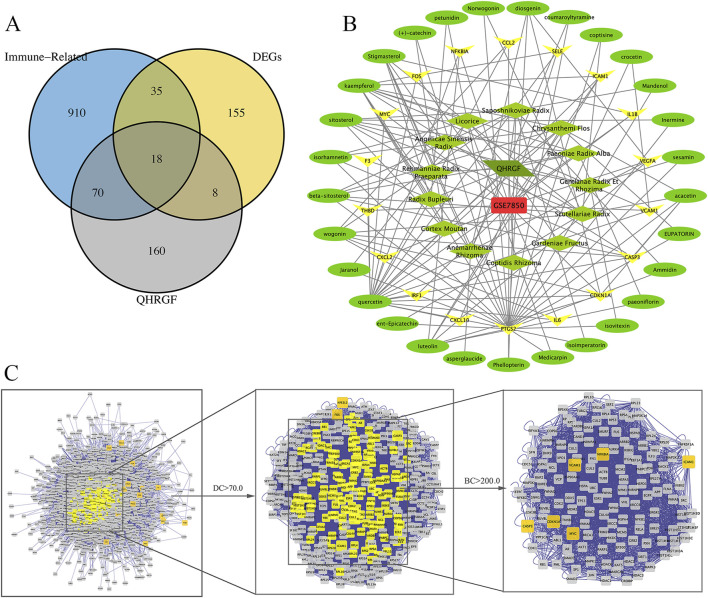
Network construction. **(A)** The Venn diagram of uveitis targets from the GSE7850 dataset and Qinghuo Rougan Formula (QHRGF) targets. **(B)** Construction of the “QHRGF-compound-target-uveitis” network. Green diamonds and circles represent herbs in QHRGF and active compounds, respectively. Yellow nodes represent target proteins. **(C)** The process of topological screening for constructing the protein-protein interaction (PPI) network. The network includes 18 overlapping targets that were shared between differentially expressed genes (DEGs) and QHRGF-predicted targets. It does not include 8 additional immune-related genes that were identified later during functional enrichment analysis. These were added in downstream analysis for pathway interpretation but are not part of the primary intersection used in network construction.

Analysis of common targets among putative targets and DEGs that are potential targets of QHRGF revealed 18 common disease-drug targets (Figure 2A). We found 18 genes that overlapped between predicted QHRGF targets and DEGs. In a later step, we also included 8 immune-related genes from pathway enrichment results. This gave a total of 26 genes. These genes were linked to inflammation-related pathways. An interactive QHRGF-compound-target-uveitis network was constructed (Figure 2B). This network comprised 70 nodes and 168 edges, suggesting the complex correlations among different compounds and targets.

The topological feature analysis of the PPI was performed using the Cytoscape plug-in CytoNCA based on the following two major parameters: DC and BC. The criterion of the first screening was DC ≥ 70, which yielded 368 targets and 14,077 edges (Figure 2C). Next, 143 targets were then further screened with a criterion of BC ≥ 200, which yielded 6 targets and 136 edges. These six targets were *CDKN1A*, *VCAM1*, *NFKBIA*, *ICAM1*, *CASP3*, and *MYC*, which can serve as the targets for the therapeutic effect of QHRGF on uveitis.

### 3.5 Functional and pathway enrichment analysis of the 18 common targets

To further explore the underlying mechanism of QHRGF in uveitis, 26 common targets were subjected to functional enrichment analyses. The top 3 enrichments of targets in the GO term BP were response to lipopolysaccharide, response to molecule of bacterial origin, and leukocyte migration. In the CC category, the shared targets were significantly associated with membrane raft and membrane microdomain. The top enrichment of shared targets in the MF category was cytokine receptor binding (Supplementary Figure S3A, B). Next, the shared genes were subjected to KEGG pathway enrichment analysis. Based on the threshold of adj-P value <0.05, 59 KEGG pathways were obtained. Thirty significant pathways significantly associated with the pathogenesis of uveitis were shown in Supplementary Figures S3C, D.

### 3.6 Weighted co-expression network construction and key module identification

**FIGURE 3:**
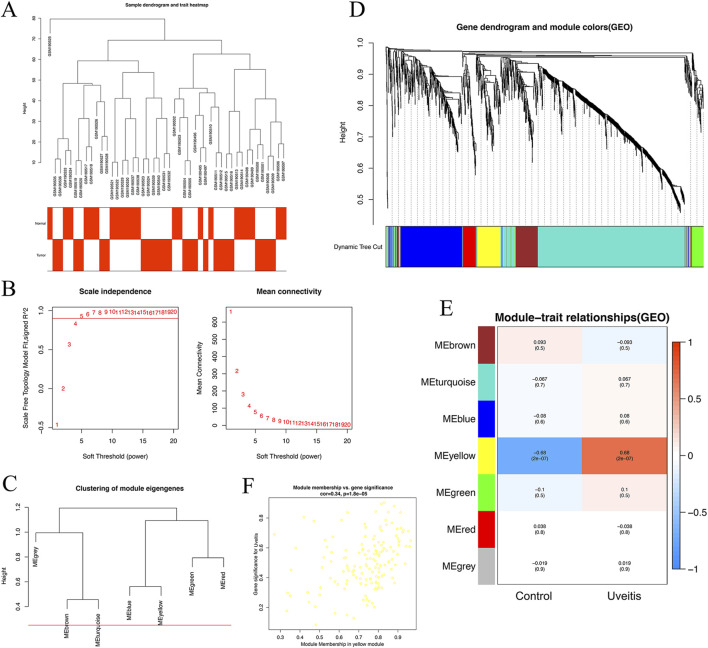
Identification of modules associated with uveitis in the GSE7850 dataset. **(A)** Sample dendrogram and trait indicator. Sample clustering did not indicate outliers. **(B)** Analysis of the scale-free fit index and the mean connectivity for various soft-thresholding powers (β). **(C)** Clustering of module eigengenes. The red line indicates the cut-off height (0.25). **(D)** The cluster dendrogram of the common differentially expressed genes based on the 1-topological overlap matrix (TOM). Each module assigned with a unique color indicates a cluster of co-expressed genes. **(E)** Heatmap of the correlation between module eigengenes and clinical traits of uveitis. **(F)** Scatter plot of module eigengenes in the yellow module.

To find the key modules associated with uveitis, all genes of 44 samples in the GSE7850 dataset were subjected to co-expression analysis with the ‘WGCNA’ package in R. After quality control using the WGCNA R package, none of the samples were removed in the sample clustering (Figure 3A). We used a soft-thresholding power (β) to highlight strong gene correlations and reduce weak ones. The β value was set to 5. At this value, the scale-free topology fitting index *R* ^2^ reached 0.90, which ensured a scale-free network (Figure 3B). We set the cut-off height to 0.25. This gave us seven modules for further analysis (Figures 3C,D). Among these, the yellow module showed the strongest link to uveitis. This was seen in the module–trait correlation heatmap (Figure 3E).We also plotted the scatterplot of gene significance (GS) for uveitis against module membership (MM) in the yellow module (Figure 3F). This module included 120 genes. The correlation with uveitis was strong (r = 0.72, p = 1.8 × 10^−5^). This result remained significant after FDR correction (FDR <0.05).The other six modules showed weaker or no significant correlations. Based on these results, we selected the yellow module for further analysis.

### 3.7 Identification of cross-referencing immune-related overlapped hub genes

**FIGURE 4:**
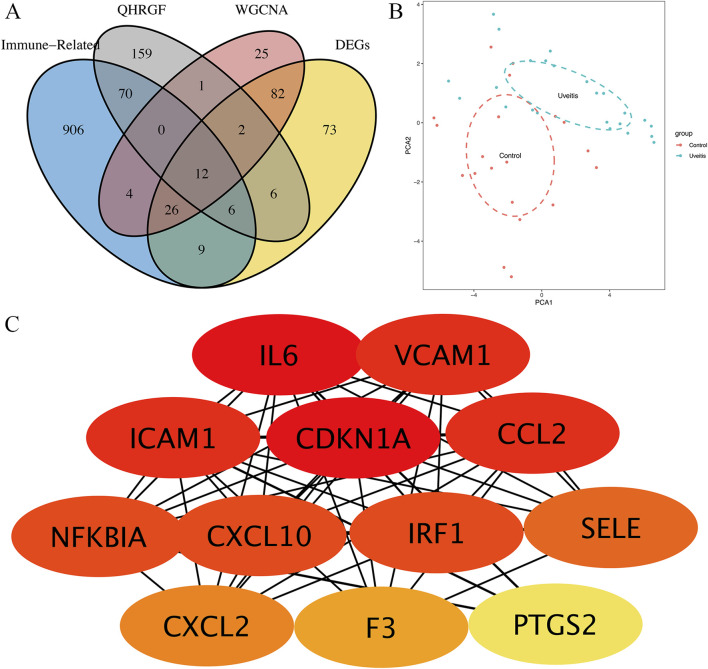
Identification of hub genes. **(A)** Twelve overlapped targets from the GSE7850 dataset, QRF, and GeneCards and weighted gene co-expression network analysis (WGCNA). **(B)** Principal component analysis revealed differential gene expression between uveitis and control samples. **(C)** The protein-protein interaction network of the 12 overlapping genes. The red nodes represent the big key nodes, followed by the orange and yellow nodes.

The Venn diagram was used to intersect the putative targets of QHRGF, DEGs, and yellow module genes. In total, 12 overlapping genes were retained in this study (Figure 4A). PCA was performed to further examine the distinct distribution between uveitis and control samples using the expression profile of 12 overlapping genes. The samples tended to be sorted into two sections. The distribution model of uveitis samples was significantly different from that of control samples (Figure 4B). In the STRING database, the PPI network of the 12 overlapping genes was constructed. These 12 overlapping targets exhibited high confidence scores (confidence score ≥0.4). In the PPI network, the disconnected nodes were hidden, indicating their strong interactions. As shown in Figure 4C, the networks were generated using the Cytoscape plug-in CytoHubba to calculate and identify the importance degree of overlapped targets.

### 3.8 Prediction of potential biomarkers using the LASSO model

**FIGURE 5:**
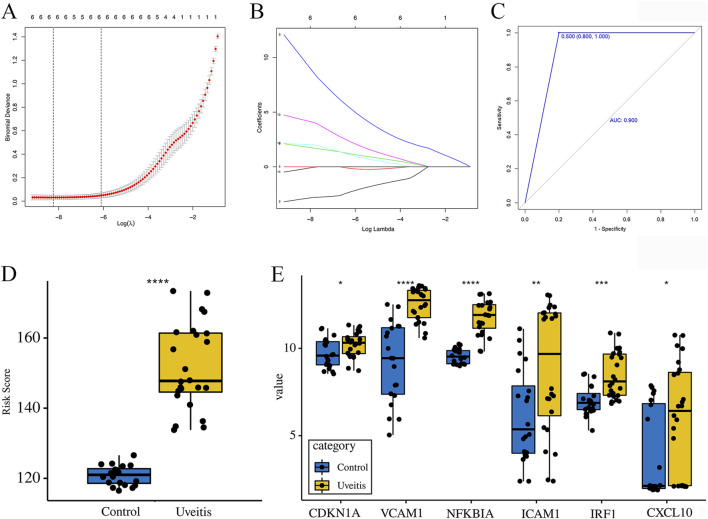
A model for predicting uveitis and verification of the expression of the model-related genes. **(A)** The optimal penalty parameter (λ) is selected using five-fold cross-validation with minimum criteria. **(B)** Least absolute shrinkage and selection operator coefficient profiles of the 12 hub genes. **(C)** The area under the curve (AUC) was 0.900. **(D)** The model index value in the uveitis samples was higher than that in the control samples. **(E)** The expression levels of the model-related genes in uveitis samples relative to those in the control samples.

The expression profiles of 12 overlapping genes were extracted to construct the LASSO model (Figures 5A,B). Based on LASSO Cox regression analysis, 6 genes (*CDKN1A*, *VCAM1*, *NFKBIA*, *ICAM1*, *IRF1*, and *CXCL10*) were retained to construct the model index. The gene-based model index was calculated as follows: model index = (*CDKN1A* × −4.5978674) + (*VCAM1* × 2.4030104) + (*NFKBIA* × 12.5086637) + (*ICAM1* × −0.2771247) + (*IRF1* × 3.3248412) + (*CXCL10* × −1.0133916). ROC curve analysis was performed to evaluate the potential diagnostic performance of the constructed prognostic model and AUC value was 0.900 (Figure 5C). The box diagram revealed that the model index values in the uveitis samples were higher than those in the control samples (Figure 5D). Furthermore, the expression levels of 6 genes in uveitis samples were higher than those in control samples (Figure 5E). These results suggest that the selected genes and the model index are highly correlated with uveitis. The cross-validated model demonstrated strong classification performance, indicating its potential value as a predictive signature.

### 3.9 GSEA

GSEA revealed that compared with those in the CDKN1A-low group, the KEGG pathways, such as the MAPK, T-cell receptor, chemokine, and apoptosis signaling pathways were significantly enriched in the CDKN1A-high group (Supplementary Figure S4). Meanwhile, the KEGG pathways, such as the MAPK and T-cell receptor signaling pathways in the VCAM1-high, ICAM1-high, NFKBIA-high, IRF1-high, and CXCL10-high groups were significantly enriched when compared with those in the VCAM1-low, ICAM1-low, NFKBIA-low, IRF1-low, and CXCL10-low groups (Supplementary Figure S4; Supplementary Figure S5).

### 3.10 Molecular docking

**FIGURE 6:**
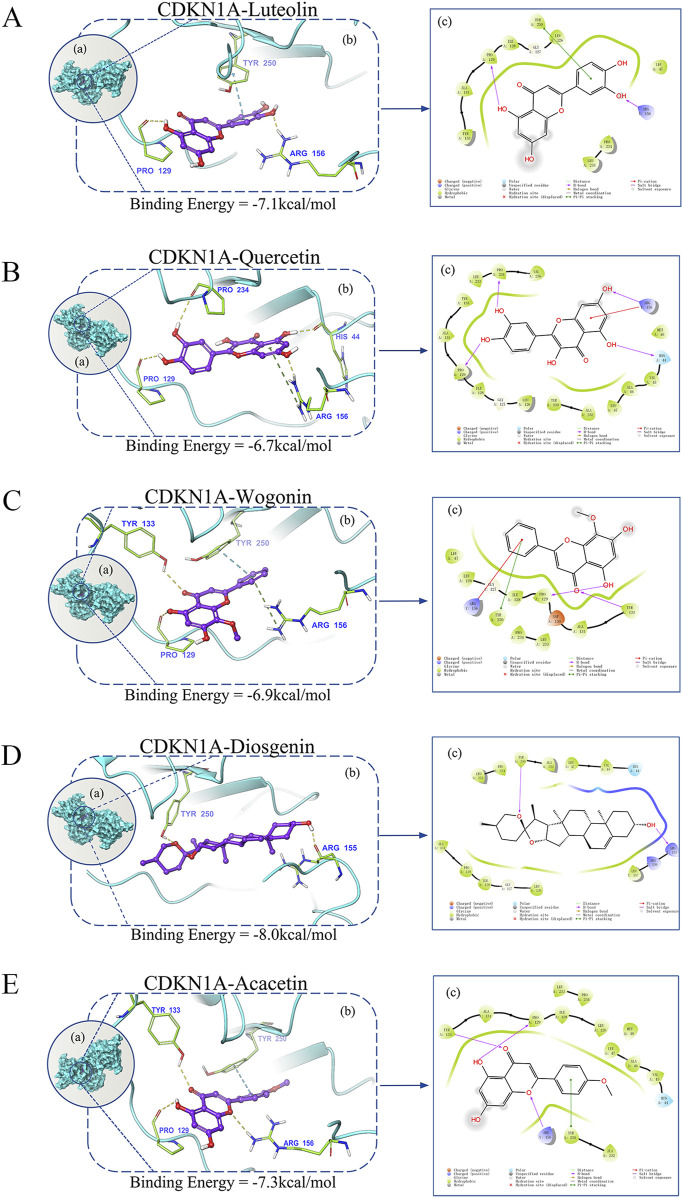
Evaluation of the binding mode of screened drugs to their targets using molecular docking. **(A–E)** Binding mode of luteolin, quercetin, wogonin, diosgenin, and acacetin to CDKN1A. **(a)** A schematic showing the overlay of the crystal structures of small molecule compounds and their targets was illustrated using the Molecule of the Month feature. **(b)** Three-dimensional structures of the binding pockets were visualized using the PyMOL software. **(c)** Two-dimensional interactions of compounds and their targets. All docking poses shown represent the top-ranked conformations with the lowest binding energy (kcal/mol) for each compound–target pair.

To clarify the mechanism of the selected six hub genes and their corresponding compounds at the molecular level, the compounds were docked to the corresponding active pockets of the target proteins. Five compounds docked to the active pockets of CDKN1A. The specific data and theory combination model are shown in Figure 6. The interaction between the protein target and the small molecule compound was mediated predominantly by hydrogen bonds. These interactions enabled the protein and the compound to form a stable complex.

### 3.11 Ocular inflammation

**FIGURE 7:**
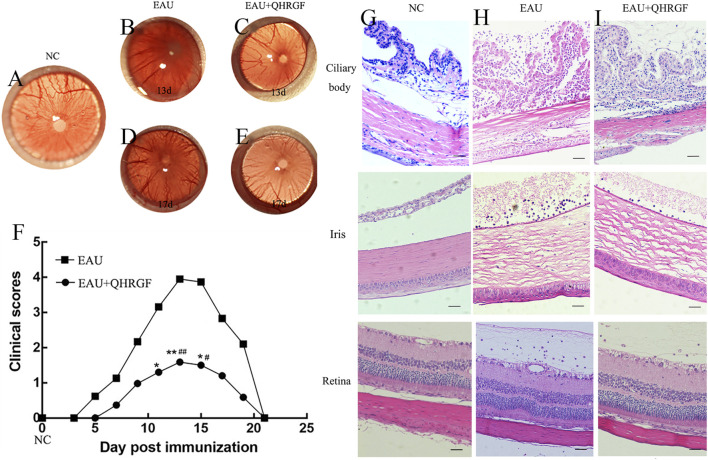
Evaluation of clinical symptoms. **(A)** The representative images of intraocular inflammation in the healthy control (NC) groups were captured using a Genesis-D camera. **(B–E)** The representative images of intraocular inflammation in the experimental autoimmune uveitis (EAU) and Qinghuo Rougan Formula (QHRGF) groups were captured using a Genesis-D camera on days 13 and 17 post-immunization. **(F)** Changes in the clinical scores of the three groups at different time points after immunization. The clinical scores are presented as mean ± standard deviation. ^*^P < 0.05, compared with the NC group; ^#^P < 0.05 compared with the EAU group. **(G–I)** Histopathological alterations in the ciliary body, iris, and retina of the three groups after immunization. The sections were subjected to hematoxylin and eosin staining. Scale bar = 50 μm. Data are shown as mean ± SD. Each group included 3 rats (n = 3).

The eye tissues of the NC group (Figure 7A) did not exhibit distension and engorgement of the iris vessels. Ocular inflammation in the EAU and QHRGF groups peaked on day 13 post-immunization. Inflammation was characterized by dilated blood vessels in the iris, fibrin-like exudate in anterior chamber, abnormal pupil contraction, and other symptoms. The severity of ocular inflammation was remarkably attenuated in the QHRGF group when compared with the EAU group (Figures 7B,D). Meanwhile, on day 17 post-immunization, the severity of inflammation was significantly mitigated in the EAU and QHRGF groups (Figures 7C,E). The clinical scores of rats in the EAU and QHRGF groups were recorded based on the clinical features at different time points (Figure 7F).

### 3.12 Histopathological analysis

Histopathological examination revealed distinct inflammatory changes across ocular tissues. In the ciliary body and iris, the EAU group showed marked immune cell infiltration, edema, and structural disorganization compared to the NC group, which exhibited normal tissue architecture with no obvious infiltration (Figures 7G,H). QHRGF treatment markedly alleviated inflammatory responses in these regions, with reduced cell infiltration and preserved morphology (Figure 7I). In the retina, a semi-quantitative histological scoring system was applied to assess inflammatory infiltration and structural damage, following previously published criteria (Agarwal et al., 2012). Rats in the NC group displayed normal retinal layering and no signs of inflammation (mean score: 0.3 ± 0.1), whereas the EAU group exhibited extensive infiltration, retinal disorganization, and tissue swelling (mean score: 3.2 ± 0.2; Figure 7H). Notably, QHRGF administration significantly reduced retinal pathology, preserving retinal structure with limited cellular infiltration (mean score: 1.5 ± 0.3; Figure 7I). Statistical analysis confirmed a significant difference between the EAU and QHRGF groups (P < 0.05).

### 3.13 The mRNA and protein levels of IL-10, IL-4, IL-17, and IFN-γ

**FIGURE 8:**
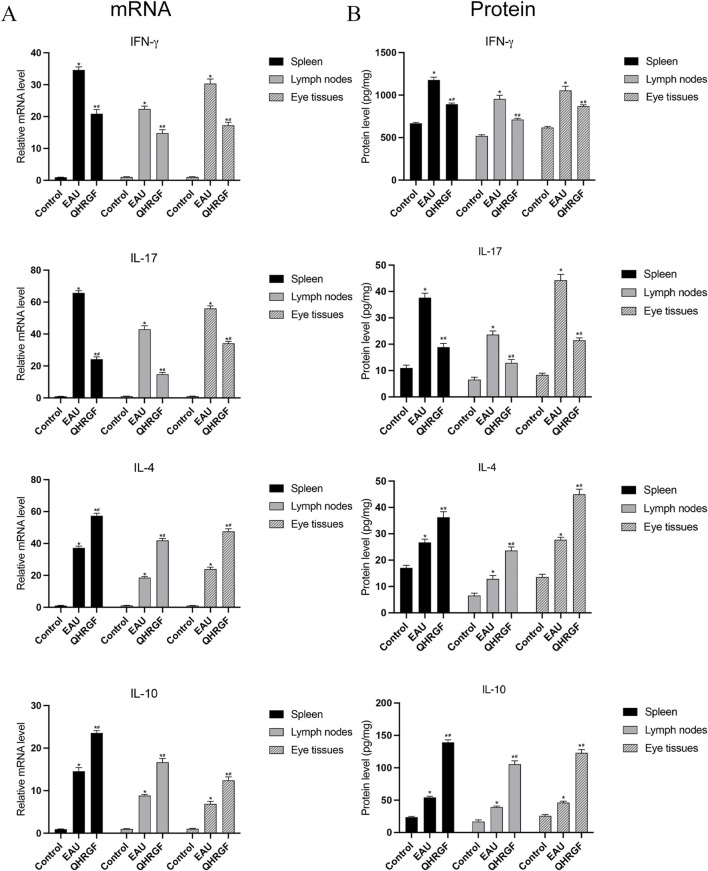
Expression of Notch1, DLL4, IL-10, and IL-17A mRNA in spleen, lymph nodes, and eye tissues from the rats in NC, EAU and LXD groups at 13 days after immunization. **(A)** mRNA levels of IFN-γ, IL-17, IL-4, and IL-10. **(B)** Protein levels of IFN-γ, IL-17, IL-4, and IL-10. ^*^P < 0.05, compared with the NC group; ^#^P < 0.05 compared with the EAU group. Data are shown as mean ± SD. Each group included 3 rats (n = 3).

Results showed, using q-PCR, that the mRNA levels of IFN-γ, IL-17, IL-4, and IL-10 in the liver tissues of the rats in the EAU group were significantly associated with the progression of uveitis (Figure 8A). On day 13 post-immunization, the QHRGF treatment caused a significant increase in the mRNA levels of IL-10, in comparison to the EAU and control groups. A similar inverse trend was also observed in IL4 levels. As for the mRNA levels of IL-17 and IFN-γ, the expression levels were significantly reduced in the QHRGF treatment group compared with the EAU group. Furthermore, the protein levels of IFN-γ, IL-17, IL-4, and IL-10 in spleen, lymph nodes and eye tissues were consistent with those of mRNA expression (Figure 8B).

## 4 Discussion

Chinese herbal formulations can significantly reduce the recurrence rate of uveitis and alleviate the side effects caused by corticosteroids or immunosuppressive agents. The identification of immune-related biomarkers is an important step in the diagnosis, prognosis, and prevention of uveitis. Additionally, the correlation and interaction between these biomarkers must be elucidated. Previously, we reported that QHRGF significantly inhibited uveitis by regulating natural killer T cells and inhibiting the MAPK signaling pathways *in vivo* (Jing et al., 2019). However, the underlying molecular mechanisms and biomarkers were not elucidated. Based on the findings of our previous study, this study performed a comprehensive network pharmacological analysis of QHRGF. The GEO dataset was examined using WGCNA. The overlapping hub genes obtained from the analysis can be potential novel biomarkers with increased specificity for the early diagnosis of uveitis.

In this study, network pharmacology methods were used to identify bioactive compounds in QHRGF and their target proteins. Next, GO and KEGG pathway enrichment analyses were performed with the 18 immune-related common targets based on the constructed QHRGF-compound-target-uveitis network. Several compounds in QHRGF have demonstrated favorable pharmacokinetic and safety characteristics. For example, baicalin and luteolin, two of the major flavonoids in the formulation, have shown moderate oral bioavailability and low systemic toxicity in preclinical studies (Hu et al., 2022; Zhang and Ma, 2024). These findings support the potential for clinical development of QHRGF-based therapies. Nonetheless, we acknowledge that additional pharmacokinetic profiling and toxicological evaluations are required to fully assess the safety and efficacy of QHRGF in a clinical setting. Functional enrichment analysis revealed that modules with a strong correlation with uveitis, including the TNF, malaria, and IL-17 signaling pathways, were consistent with the findings of our previous study. WGCNA, a systems biology method, determines the correlation between the clinical traits and modules using the optimal soft-threshold power (Qian et al., 2020). Weighted network methods are useful for identifying consensus modules as they enable the calibration of individual networks (Horvath et al., 2012). In the present study, WGCNA of the uveitis dataset (GSE7850) revealed seven functional modules. The yellow module was highly correlated with uveitis. After intersection, 12 cross-referencing overlapped genes with high functional significance in the QHRGF-compound-target-uveitis network and the yellow weighted network module were obtained. In the PPI network, the level of importance of these overlapping genes was determined and visualized using ‘Cytohubba.’ The top 3 genes were *IL6*, *VCAM1*, and *ICAM1*.

*CDKN1A*, *VCAM1*, *NFKBIA*, *ICAM1*, *IRF1*, and *CXCL10* were then identified with non-zero regression coefficients in the LASSO model. LASSO regression was selected over other machine learning methods, such as Random Forest or XGBoost, due to its strength in producing interpretable models with a minimal set of non-zero predictors. In contrast to ensemble methods, LASSO offers direct insights into the contribution of individual genes to the model (Tibshirani, 1996). ROC curve analysis demonstrated the LASSO model represented a high AUC value (AUC = 0.9), suggesting that this model may act as a biomarker for uveitis. The most important hub genes were similar in topology analysis of the 18 common targets, the PPI network, and the constructed LASSO model. This finding is consistent with that of Becker. et al. (Becker et al., 2012) who reported that multi-clustered proteins are central in the network, contain increased numbers of domains, and are involved in several regulatory processes. These features are considered hallmarks of multifunctional proteins. Notably, several genes in the panel are known to be expressed in peripheral immune cells and detectable in blood or aqueous humor during ocular inflammation. This raises the possibility that the model could be further developed for non-invasive clinical applications, such as early diagnosis or disease monitoring of uveitis.

Functionally, these six hub genes converge on key inflammatory and immune pathways implicated in uveitis, including NF-κB, MAPK, TNF, and chemokine signaling. *CDKN1A* is a cyclin-dependent kinase inhibitor and direct transcriptional target of NF-κB and p53. It mediates cell cycle arrest in response to stress and inflammation, and has been shown to suppress T cell- and macrophage-mediated inflammatory responses via MAPK pathway modulation (Perkins, 2007; Seo et al., 2011). *VCAM1* encodes a vascular adhesion molecule upregulated by TNF-α and IL-1β, facilitating leukocyte adhesion and transmigration across the endothelium. It has been strongly associated with retinal vascular inflammation and uveitis pathogenesis (Yousef et al., 2019). *NFKBIA* encodes IκBα, which inhibits nuclear translocation of NF-κB. Its degradation releases NF-κB, promoting transcription of inflammatory mediators such as cytokines, chemokines, and adhesion molecules including *ICAM1* (Hoffmann et al., 2002). *ICAM1* is another adhesion molecule induced by inflammatory stimuli. Its upregulation in retinal endothelial cells has been linked to blood-retinal barrier dysfunction and leukocyte infiltration in autoimmune eye diseases (He et al., 2014). *IRF1* is a transcription factor activated downstream of type I/II interferon and Toll-like receptor pathways. It enhances expression of proinflammatory genes including *CXCL10* and has been implicated in Th1-dominant autoimmune inflammation (Taniguchi et al., 2001). *CXCL10* is a chemokine that attracts activated T cells via CXCR3. Elevated levels have been observed in aqueous humor of uveitis patients and in experimental autoimmune uveitis (EAU) models, where it contributes to CD4^+^ T cell recruitment and tissue damage (Curnow et al., 2005). These functional roles are in agreement with our GSEA findings, where the six genes were enriched in MAPK, chemokine, and T-cell receptor signaling pathways. The overlap between transcriptomic, network, and machine learning analyses supports the robustness of these genes as key inflammatory mediators in uveitis. Given their mechanistic relevance and potential detectability in body fluids (e.g., *CXCL10*, *ICAM1*), this panel may serve as a basis for future diagnostic or therapeutic exploration.

Molecular docking simulation has important applications in the field of computer-aided drug design and is often used to explain potential intermolecular interactions (Xue et al., 2020). This study demonstrated that five compounds dock to CDKN1A, indicating the advantages of QHRGF with multiple components and multiple targets in treating uveitis. Molecular docking study provided a reasonable explanation for the interaction between proteins and compounds and further confirmed the effectiveness and specificity of QHRGF in the treatment of uveitis. This study used network pharmacology analysis because the final targets identified using this method can not only act as markers for the early diagnosis of uveitis but also as therapeutic targets.

In conclusion, this study assessed the targets of QHRGF involved in its therapeutic effects on uveitis using systems biology methods, including WGCNA and network pharmacology. This study identified six significantly upregulated genes (*CDKN1A*, *VCAM1*, *NFKBIA*, *ICAM1*, *IRF1*, and *CXCL10*) and demonstrated that QHRGF exerts therapeutic effects on uveitis using *in vivo* experiments. However, future studies must examine the contribution of the regulatory effects of these six hub genes to uveitis development.

## Data Availability

The datasets presented in this study can be found in online repositories. The names of the repository/repositories and accession number(s) can be found in the article/Supplementary Material.
