# Supplementary material for: Integrated network pharmacology and in vivo experiments to reveal the anti-inflammatory mechanism of Qinghuo Rougan Formula in uveitis
Source: Front Mol Biosci. 2025 Jul 11;12:1632027. doi: 10.3389/fmolb.2025.1632027 (PMC12289509; doi:10.3389/fmolb.2025.1632027)
Supplement: Supplementary file 1 [file Supplementaryfile1.docx]

**Supplementary Figures**

**
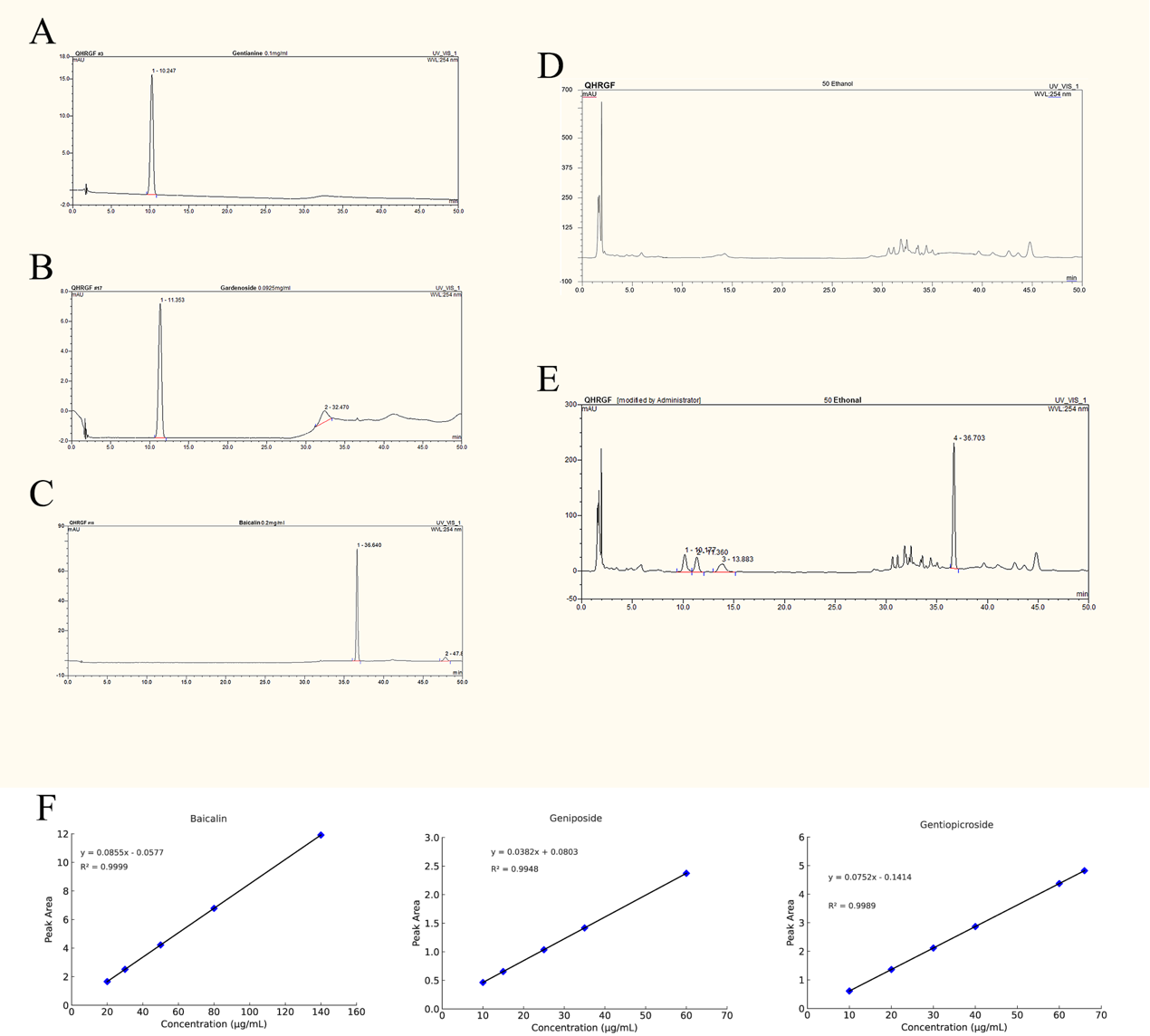
**

**Fig. S1.** High-Performance Liquid Chromatography Analysis of QHRGF. (A-C) represent the peaks of the three main active compounds. (D) represents the negative control group of QHRGF. (E) represents the HPLC results of the positive sample containing the three main compounds. (F) The linearity was established over appropriate concentration ranges


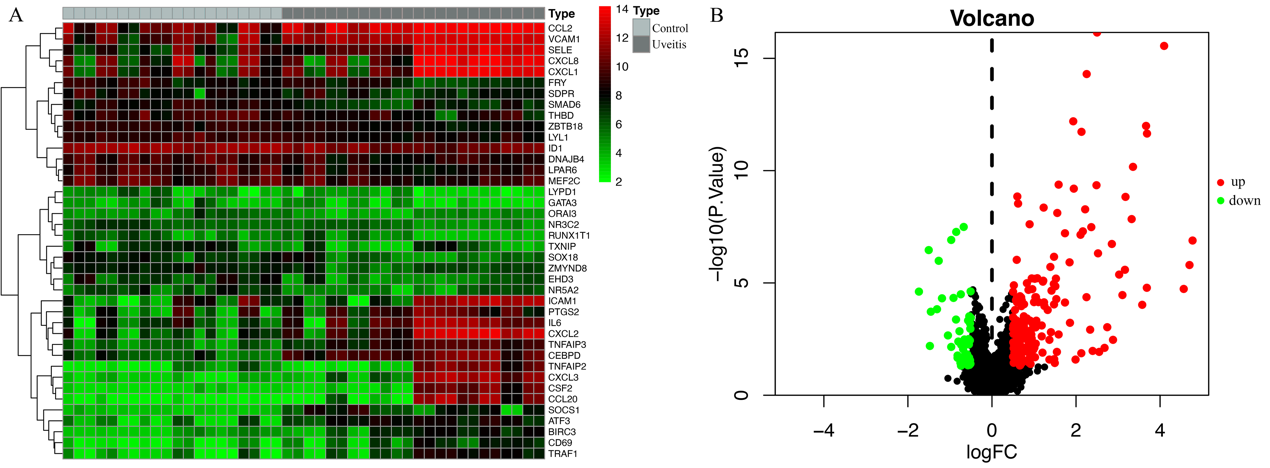


**Figure S2.** Identification of differentially expressed genes (DEGs) between uveitis and control samples. (A) Heatmap of DEGs in the GSE7850 dataset. (B) Volcano plot of DEGs in the GSE7850 dataset. Red and blue dots represent upregulated and downregulated genes, respectively. Black dots represent non-significant genes.


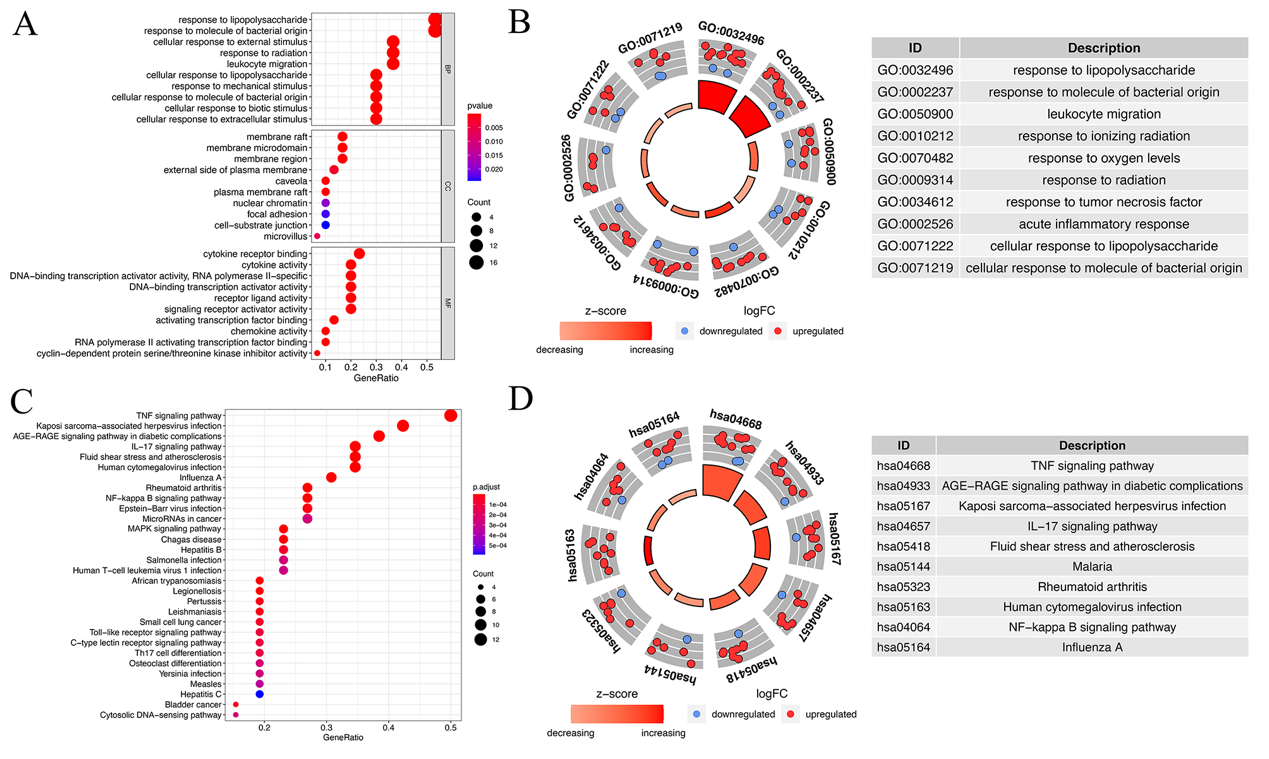


**Fig. S3.** Gene Ontology (GO) and Kyoto Encyclopedia of Genes and Genomes (KEGG) functional annotation analyses of 18 differentially expressed ferroptosis-related genes. (A) Bubble chart of significantly enriched GO terms (biological process (BP), cellular component (CC), and molecular function (MF)). (B) The circle shows the scatter map of the specified gene in each GO term. The red and blue circles represent upregulated and downregulated genes, respectively. The Z-score is directly proportional to the expression of the enriched pathway. (C) Bubble chart of KEGG pathways. (D) The circle shows the scatter map of the KEGG pathway. The red and blue circles represent upregulated and downregulated genes, respectively. The Z-score value is directly proportional to the expression of the enriched pathway.


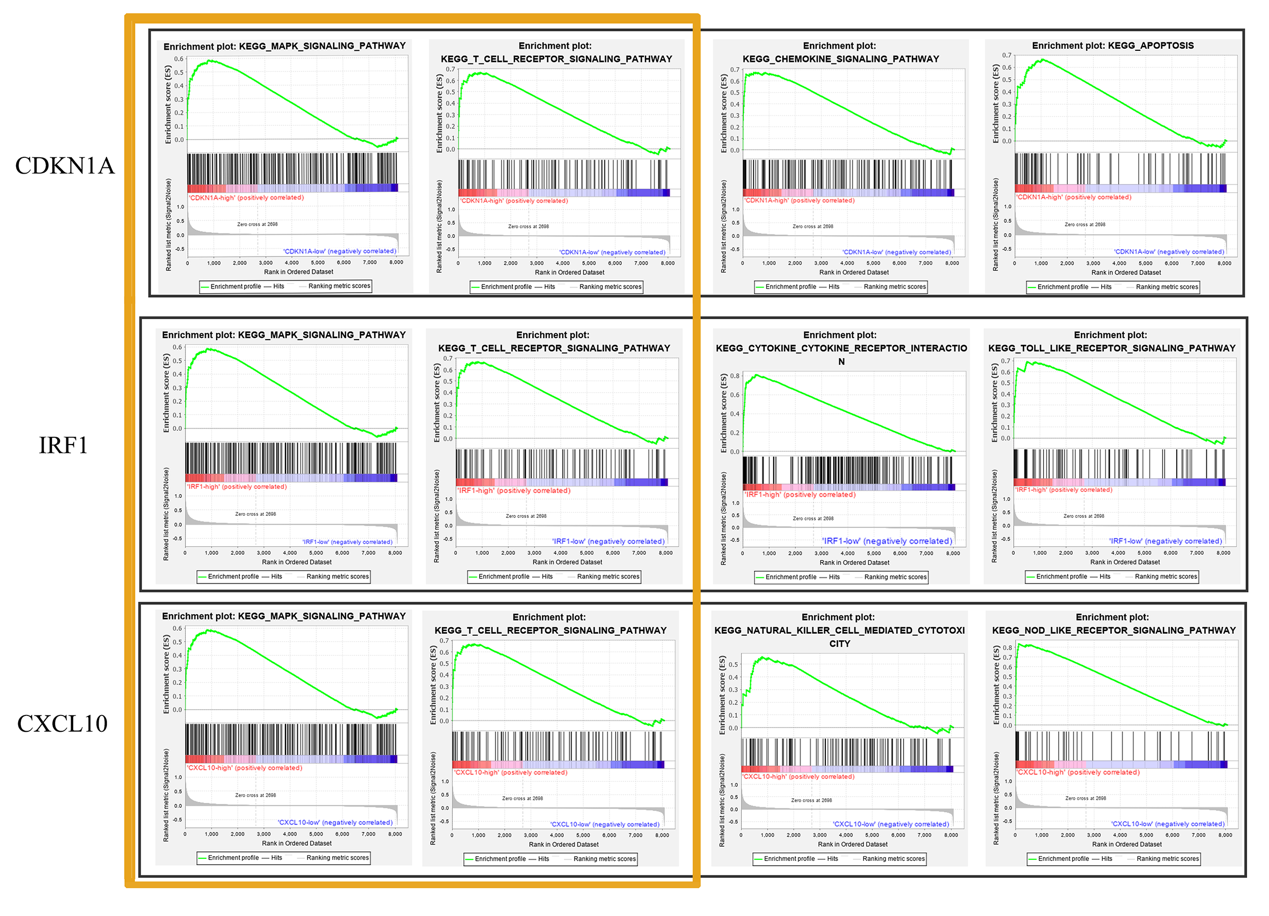


**Figure S4.** Gene Set Expression Analysis (GSEA). Kyoto Encyclopedia of Genes and Genomes (KEGG) pathways in which *CDKN1A*, *IRF1*, and *CXCL10* are enriched.


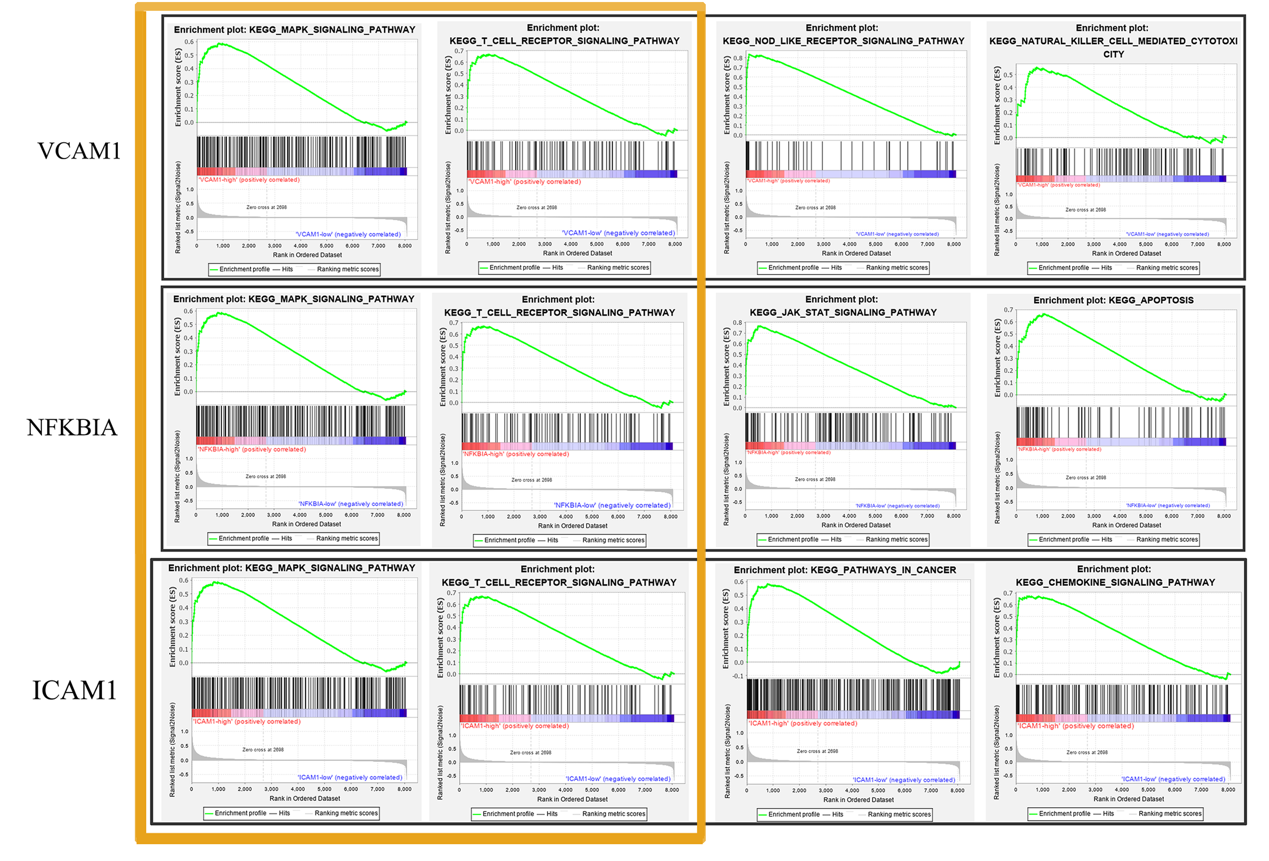
 **Figure S5.** Gene Set Expression Analysis (GSEA). Kyoto Encyclopedia of Genes and Genomes (KEGG) pathways in which *VCAM1*, *NFKBIA*, and *ICAM1* are enriched.
